# Activated Iron-Porous Carbon Nanomaterials as Adsorbents for Methylene Blue and Congo Red

**DOI:** 10.3390/molecules29174090

**Published:** 2024-08-29

**Authors:** Daniel Sibera, Iwona Pełech, Piotr Staciwa, Robert Pełech, Ewa Ekiert, Gulsen Yagmur Kayalar, Urszula Narkiewicz

**Affiliations:** 1 Department of General Civil Engineering, Faculty of Civil and Environmental Engineering, West Pomeranian University of Technology in Szczecin, al. Piastów 50a, 70-311 Szczecin, Poland; 2 Department of Chemical and Environment Engineering, Faculty of Chemical Technology and Engineering, West Pomeranian University of Technology in Szczecin, Pułaskiego,10, 70-322 Szczecin, Poland; piotr.staciwa@zut.edu.pl (P.S.); ewa.dabrowa@zut.edu.pl (E.E.); urszula.narkiewicz@zut.edu.pl (U.N.); 3 Department of Chemical Organic Technology and Polymeric Materials, Faculty of Chemical Technology and Engineering, West Pomeranian University of Technology in Szczecin, Pułaskiego 10, 70-322 Szczecin, Poland; robert.pelech@zut.edu.pl; 4 Department of Chemical Engineering, Faculty of Engineering, Eskişehir Technical University, 26555 Eskişehir, Turkey; gulsenyagmurr@gmail.com

**Keywords:** carbon spheres, microwaves, dyes, methylene blue, congo red, adsorption

## Abstract

The adsorption properties of microporous carbon materials modified with iron citrate were investigated. The carbon materials were produced based on resorcinol-formaldehyde resin, treated in a microwave assisted solvothermal reactor, and next carbonized in the tube furnace at a temperature of 700 °C under argon atmosphere. Iron citrate was applied as a modifier, added to the material precursor before the synthesis in the reactor, in the quantity enabling to obtain the nanocomposites with C:Fe mass ratio equal to 10:1. Some samples were additionally activated using potassium oxalate or potassium hydroxide. The phase composition of the produced nanocomposites was determined using the X-ray diffraction method. Scanning and transmission electron microscopy was applied to characterize the changes in samples’ morphology resulting from the activation process and/or the introduction of iron into the carbon matrix. The adsorption of nitrogen from gas phase and dyes (methylene blue and congo red) from water solution on the obtained materials was investigated. In the case of methylene blue, the adsorption equilibrium isotherms followed the Langmuir isotherm model. However, in the case of congo red, a linear dependency of adsorption and concentration in a broad equilibrium concentration range was found and well-described using the Henry equation. The most efficient adsorption of methylene blue was noticed for the sample activated with potassium hydroxide and modified with iron citrate, and a maximum adsorption capacity of 696 mg/g was achieved. The highest congo red adsorption was noticed for the non-activated sample modified with iron citrate, and the partition coefficient for this material equaled 171 dm^3^/g.

## 1. Introduction

Even small amounts of dyes in the water are undesirable. Dyes give water an unsightly appearance and interfere with the life processes taking place in the water. Currently, over 100,000 types of dyes are used in the industry, and their annual production reaches 70 thousand tones [1,2]. About 10–15% of organic dyes used in textile and other industries like rubber, paper and printing, plastic, cosmetics, leather, pharmaceuticals, and food are discharged in the surrounding ecosystem. Such a high presence of dyes in water and sewage poses a serious threat to aquatic organisms, animals, and humans. Efficient treatment of colored wastewater is very important to alleviate environmental pollution. 

The most common methods of removing dyes from wastewater are: membrane separation [3,4], coagulation [5], ozonation [6], photocatalysis [7], and adsorption [8,9]. The use of most of these methods is associated with high operating and production costs. However, the adsorption process has been considered as a most effective and reliable method for dye removal due to its easy operation, low cost, recyclability, and high efficiency. Various carbon materials e.g., graphene-based composites [10,11,12], carbon nanotubes [13,14,15,16], and porous carbon [17,18], are widely used as adsorbents in wastewater treatment that allow the adsorption of dyes. Activated carbon is the most used adsorbent due to the large surface area and high porosity; it has a high adsorption capacity [19,20,21,22]. However, carbon materials for dye adsorption also have disadvantages. The separation and recovery of carbon materials from solution after using is difficult, which limits their applications. This is clearly visible for carbon materials with small particle sizes. Magnetic carbon materials can be easily separated from the aqueous solution under an external magnetic field, so the new type of magnetic carbon adsorbent could be used to solve this problem. The composite based on carbon materials and magnetic particles possess high adsorption capacity. Many studies on the combination of carbon materials and magnetic particles have been conducted. Foroutan et al. [23] used activated carbon modified with Fe_3_O_4_ to remove the cationic dye of crystal violet (CV) from aqueous solutions. A maximum adsorption capacity of 23.6 and 35.3 mg/g was obtained for activated carbon and activated carbon/Fe_3_O_4_, respectively, indicating superior adsorption capacity of Fe_3_O_4_ nanoparticles. Manippady et al. [18] adsorbed methylene blue (MB) and congo red (CR) on iron-carbon adsorbents. The CF2 adsorbent used by the authors showed the maximum adsorption capacity of 531.9 mg/g for CR and 185.2 mg/g for MB, respectively. Wang et al. [24] synthesized magnetic-activated carbon nanospheres (MACSs) using hydrothermal and pyrolysis methods. Batch tests for methylene blue removal show that an excellent adsorption capacity of 192.64 mg/g at 298 K can be obtained on these samples. Jiang et al. [25] successfully prepared magnetic sugarcane bagasse-activated carbon (MSBAC) by a simple microwave method. The adsorption results showed that the maximum adsorption capacity was 36.14 mg/g, and the pH had no significant effect on the methylene blue adsorption in the range of 2–10. Siyasukh et al. [26] showed a convenient method for the preparation of magnetic hierarchical porous carbon (MHPC) spheres, which possessed high capability for methyl orange adsorption from aqueous solution. The spheres were fabricated by water-in-oil emulsification coupled with sol-gel polymerization of resorcinol containing ferric (III) nitrate and formaldehyde to make spherical carbon precursors. The prepared precursors were further converted to carbon spheres by carbonization with nitrogen or activation with carbon dioxide, by which either micro- or mesopores could be generated within the macroporous frameworks. The magnetic hierarchical porous carbon spheres obtained by CO_2_ activation possessed tremendous adsorption capacities, as high as 1522.6 mg/g, for methyl orange removal, and were easily separated from the suspension by using an external magnetic field. As shown, the composites based on carbon and magnetic particles can be used as excellent materials for dye adsorption. They are characterized by unique chemical and physical properties: a high specific surface area, large pore volume, well-defined pore size distribution, chemical stability, and magnetic properties. 

We recently investigated [27] magnetic properties of the composites containing various iron compounds and highly microporous carbon spheres. Iron citrate, nitrate, and chloride were used to prepare samples, and the obtained products contained iron, iron carbide, or magnetite. The conclusion from the paper was that all the produced samples were characterized by high porosity and good magnetic properties. Then, thanks to the coupling of the high porosity of carbon spheres with magnetic properties of iron compounds, a potential application of the composites to removal of impurities from water, followed by a magnetic separation of the sorbent, can be performed. 

In this study, we present the preparation of carbon spheres/iron composites using a resorcinol-formaldehyde resin as a carbon source and iron citrate in a solvothermal reactor heated with microwaves. The influence of the activation with potassium oxalate and potassium hydroxide, as well as the modification with iron citrate on the physicochemical properties of the obtained materials, and the adsorption capacity was investigated. The adsorptive removal of congo red and methylene blue dye from aqueous solution onto obtained carbon materials has been discussed. 

## 2. Results and Discussion

**Figure 1:**
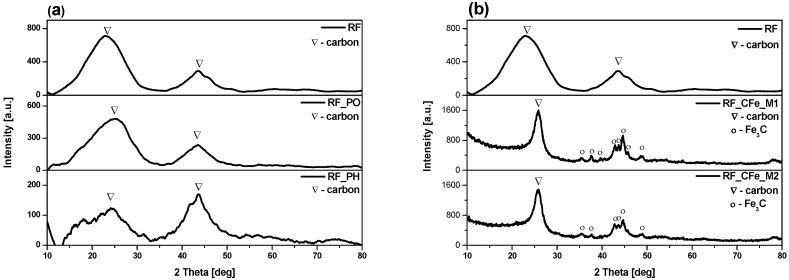

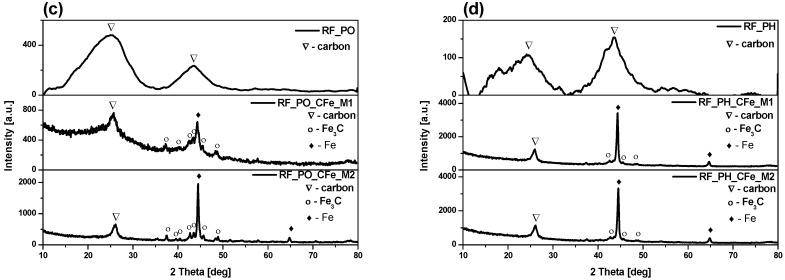
Diffraction patterns of (**a**) the reference material, (**b**) non-activated materials modified with Fe, (**c**) activated with potassium oxalate and modified with Fe, (**d**) activated with potassium hydroxide and modified with Fe.

The phase composition of the obtained materials was examined using X-ray diffraction method (XRD). The X-ray diffraction patterns of the reference samples are presented in Figure 1a and exhibited two diffraction peaks at approximately 24° and 43° 2θ, which can be assigned to (002) and (100) planes [28]. These peaks can be assigned to carbon (ICDD 00-041-1487) [29]. If carbon materials have some degree of graphitization or contain crystalline carbon phases such as graphite or graphene, then their XRD pattern may show broad diffraction peaks corresponding to the ordered carbon structures. These peaks would typically appear around 2θ values of 20–30° for graphitic carbon. The reference samples mainly contain graphitic carbon with broad peaks, indicating low graphitization and some amorphous carbon [30,31]. The XRD spectra of the non-activated materials modified with iron citrate are shown in Figure 1b. Apart from carbon, peaks corresponding to iron carbide in the form of cementite (Fe_3_C) were identified (ICDD 00-006-0688). The formation of Fe_3_C is explained by the thermal reduction process, which involves the dehydration of Fe(OH)_3_, the progressive reduction of iron oxides, and the final reaction of metallic iron with carbon at around 700 °C to form iron carbide [32]. The XRD spectra of the samples activated with potassium oxalate and potassium hydroxide and modified with iron citrate are shown in Figure 1c,d, respectively. All the activated materials show the peaks corresponding to metallic iron (ICDD 01-087-0721), carbon (ICDD 00-041-1487) and iron carbide (ICDD 00-006-0688). The presence of metallic iron in the activated samples is probably due to the decomposition of organic compounds containing iron in their structure [32]. It should be underlined that in the case of potassium hydroxide-activated samples, only small amounts of cementite were noticed in comparison to the potassium oxalate-activated samples.

**Figure 2:**
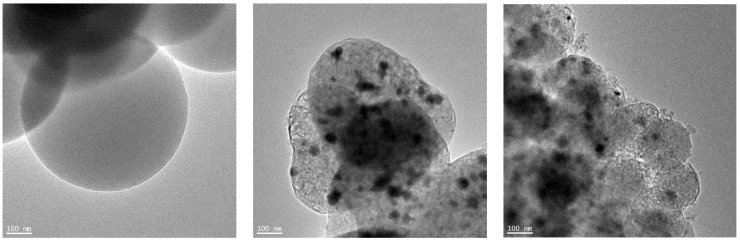

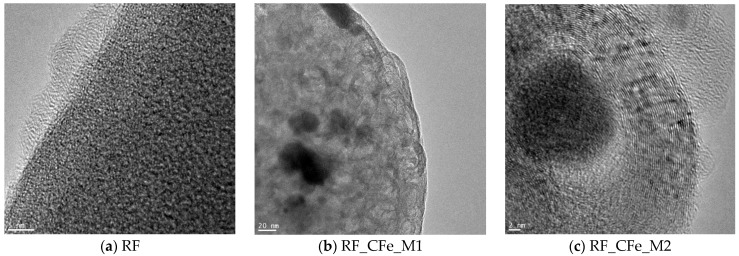
TEM images of the reference non-activated sample RF (**a**) and the non-activated samples modified with iron citrate using method 1 (**b**) and method 2 (**c**).

Transmission Electron Microscope (TEM) was used to investigate the morphology of the non-activated materials. The TEM images are shown in Figure 2. The image of the reference sample named RF presented in Figure 2a shows that the carbon structure of the spheres is amorphous, indicating a low graphitization degree [33]. In the case of carbon materials modified with iron citrate (Figure 2b,c), iron particles were distributed erratically in carbon matrix. The average size of the iron particles was about 20 nm. For the sample in which iron hydroxide was precipitated before the preparation of resorcinol-formaldehyde resin (M2), graphitic layers around the iron particles can be noticed. This phenomenon is well-known as a catalytic graphitization of carbon [34]. Incorporation of iron during the synthesis can lead to graphitization of the carbon structures. Such structures were recognized as onion-like graphitic carbon shells [35]. The orientation of the growing condensed layers visible in Figure 2c can be a result of the formation of donor-acceptor complexes with electrons originating from condensed aromatic structures of carbon [36]. 

**Figure 3:**
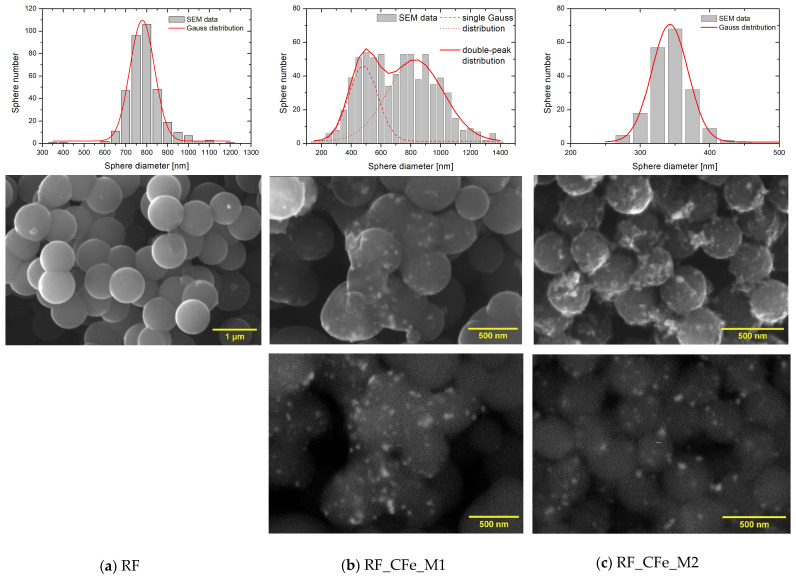
SEM images of the reference non-activated sample RF (**a**) and the non-activated samples modified with iron citrate using method 1 (**b**) and method 2 (**c**).

Scanning electron microscopy (SEM) analysis indicated that carbon spheres obtained in a standard way (RF) are well-structured, perfectly spherical, and without apparent defects (Figure 3a). A size distribution performed on the population of over 200 pcs has shown the mean diameter of ca. 780 ± 2 nm, and 99.7% of sphere diameter is within the range 606–954 nm (sigma = 58 nm). After modification of carbon material with iron, according to method M2, the sphere diameter decreased significantly to the value of 342 ± 1 nm. The resulting size distribution is even narrower, and for sigma = 26 nm the minimum and maximum diameter is 264 and 420 nm, respectively. The spheres are still well-structured, but their surface is randomly ordered and very densely decorated with particles containing iron (bright points in Figure 3c done with technique of BSE (Back Scattered Electrons). Iron concentration determined by the EDXS (Energy Dispersive X-ray Spectroscopy) method was 10.69 ± 0.41%. Enriching with iron, according to M1, introduces to the surface of carbon spheres 4.74 ± 1.35% of Fe. The procedure leads to the formation of spheres, which have diameters that vary from 200 to 1400 nm. This inhomogeneity of obtained material is clearly confirmed by the size distribution, which has a bimodal course. Size distribution can be described with two wide Gaussian distributions with two mean diameters, 481 ± 14 nm and 830 ± 24 nm. Some spheres are still connected with one another via carbon material. 

**Figure 4:**
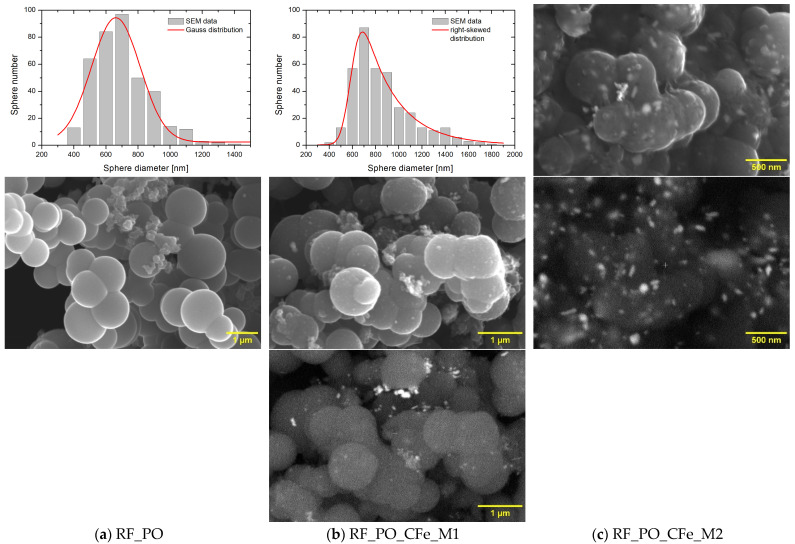
SEM images of the reference-activated potassium oxalate sample RF_PO (**a**) and the activated samples modified with iron citrate using method 1 (**b**) and method 2 (**c**).

Activation of carbon spheres using potassium oxalate leads to the formation of material where double, triple, and even more spheres are not separated during the formation, which can be seen in Figure 4 for unmodified and modified material. The diameter of the spheres varies from ca. 400 to 1500 nm, for unmodified RF_PO and modified material (M1, M2), but the mean diameter for RF_PO is 592 ± 9 nm, while for M1 it is 662 ± 49 nm. The latter size distribution follows the Gaussian model, while in the case of the M2 method the distribution shows positive/right-asymmetry, with an elongated arm in the high value side. Enriching with iron citrate using M2 leads to the formation of spheres, but they were not separated from the carbon matrix, in which iron particles are also embedded. For this sample it was impossible to make a size distribution. Both methods (M1 and M2) allowed for the introduction of comparable amounts of iron into the material, 5.85 ± 0.82% and 6.22 ± 0.88%, respectively.

Nevertheless, it can be seen from the comparison of Figure 4b,c, that in the latter case more well-dispersed on the surface bright iron nanocrystals can be observed. Then, in the case of the M2 method, more iron is on the sample surface, while for M1 iron is distributed in the bulk and on the surface.

**Figure 5:**
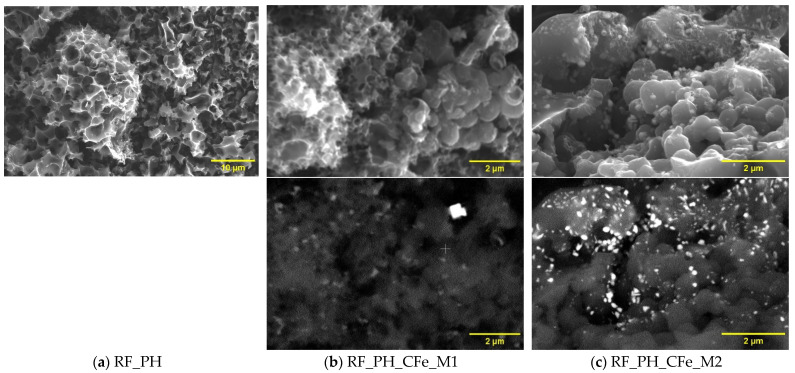
SEM images of the reference-activated potassium hydroxide sample RF_PH (**a**) and the activated samples modified with iron citrate using method 1 (**b**) and method 2 (**c**).

Activation with KOH leads to the formation of carbon material in the form of a sponge (Figure 5). This structure is visible in the reference material and after enrichment with iron as well. Some spherical structures can be found, but even they are porous. After enrichment with iron according to the M1 method, a sample containing iron concentration 2.27 ± 1.18% forms. The M2 method is allowed to introduce 10.92 ± 1.41% of iron (ca. five times greater than M1). Iron particles are embedded in carbon matrix, similar for sample RF_PO_CFe_M2.

**Figure 6:**
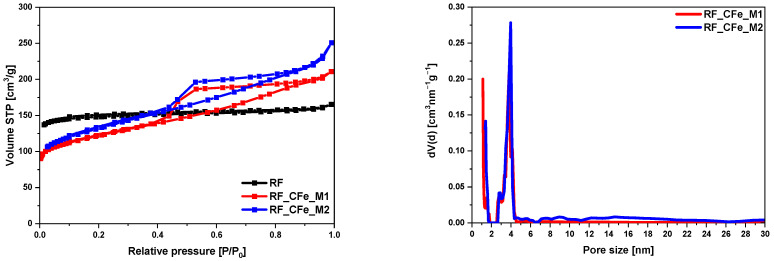
Nitrogen adsorption-desorption isotherms and the pore size distributions of iron-modified, non-activated samples.

Low-temperature (−196 °C) nitrogen adsorption measurements were used to evaluate the specific surface area and porosity of the obtained composites. The specific surface area (S_BET_), total pore volume (TPV), volume of micropores (V_m_ < 2 nm), and volume of mesopores (V_meso_) of the obtained materials were determined. The calculated results are shown in Table 1. In Figure 6 the N_2_ adsorption-desorption isotherms of the non-activated samples are visible. RF samples show type I physisorption isotherm, which corresponds to microporous materials [37,38]. For this material only a small volume of mesopores was noticed (0.04 cm^3^/g). The larger volume of mesopores was found for the samples modified with iron citrate. The volume of mesopores equaled 0.20 cm^3^/g and 0.25 cm^3^/g for RF_CFe_M1 and RF_CFe_M2, respectively. After modification with iron citrate, the shape of N_2_ adsorption isotherms was changed, what suggests significant alteration in pore structure due to iron citrate treatment. The nitrogen adsorption/desorption isotherm of these samples exhibited type II isotherms. It indicates the presence of both micropores and mesopores. Moreover, hysteresis loops H2 were observed in these samples which indicates that the pore types are various, and the pore shape is neck-like, wide, or ink bottle-like [37,39]. Such loops are often associated with aggregated crystals of zeolites and mesoporous carbons, suggesting similar structural characteristics in the modified samples [37]. As shown in Figure 6, the modification process introduced both micropores and mesopores, with a notable presence of mesopores ranging from 2.5 nm to 4.5 nm. The specific surface areas of the non-activated samples range from 400 to 455 m^2^/g. The RF sample had the largest S_BET_ equaled 455 m^2^/g. After modification with iron citrate, S_BET_ of the obtained samples decreased and equaled 400 m^2^/g and 439 m^2^/g for RF_IC_M1 and RF_IC_M2, respectively. Despite a slight reduction in the specific surface area, the total pore volume increased, particularly due to the formation of mesopores (Table 1). For the material modified using M1 and M2 method, TPV was 0.33 cm^3^/g and 0.39 cm^3^/g, respectively.

**Figure 7:**
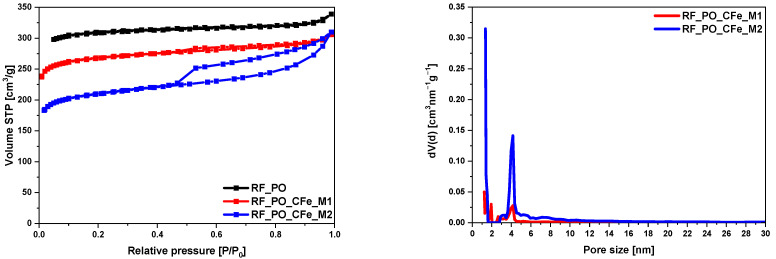
Nitrogen adsorption-desorption isotherms of activated samples with potassium oxalate and the pore size distributions of the modified samples.

In samples activated with potassium oxalate, a very similar relationship could be observed. The data in Table 1 show that the iron salt-modified samples had lower S_BET_ and TPV values compared to the RF_PO sample. As can be seen in Figure 7, the unmodified activated carbon material RF_PO and modified with the M1 method expressed the type I nitrogen isotherm. Type I isotherms indicate that these samples are primarily microporous with minimal mesopore content. The M2 modification method introduced a significant amount of mesopores, changing the pore structure to include both micropores and mesopores. In these samples we observed type II of adsorption isotherm with an H4-type hysteresis loop. Type II isotherms with an H4 hysteresis loop (M2-modified sample) indicate a significant presence of both micropores and mesopores. A sharp increase at low relative pressure (P/P_0_ < 0.01) confirms the presence of micropores. An increase from P/P_0_ 0.45 to 0.99 with a large hysteresis loop indicates the existence of mesopores [40]. The H4 hysteresis loop is indicative of narrow slit-like mesopores, suggesting a different pore structure compared to the type I isotherm samples [37]. The pore size distribution of these samples is presented in Figure 7. We observed significant mesopore content ranging from 2.8 nm to 11 nm, altering the pore structure to include a substantial amount of mesopores in the M2-modified sample. The M1-modified sample had a lower mesopore content, approximately half of the M2-modified sample.

**Figure 8:**
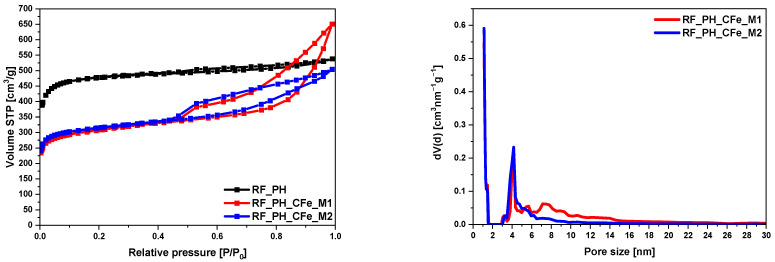
Nitrogen adsorption-desorption isotherms of activated samples with potassium hydroxide and the pore-size distributions of the modified samples.

The sample activated with potassium hydroxide RF_PH had the largest specific surface area of all tested materials, 1473 m^2^/g, resulted from a large volume of micropores 0.67 cm^3^/g and a large total pore volume 0.83 cm^3^/g. The shape of the nitrogen adsorption isotherm confirmed the microporous nature of this sample. Figure 8 presents type I of the N_2_ adsorption isotherm for this sample. Iron citrate-modified samples showed a significant increase in mesopore volume, reflected in the type II isotherm and the hysteresis loop. The H4-type hysteresis loop (sample modified using the M2 method) and a mixed type of hysteresis loop composed of H2 and H4 type (sample modified method M1) was noticed [41]. In both samples, two typical regions can be observed: first, a sharp increase at low relative pressure (P/P_0_) <  0.01 confirming the presence of micropores; second, when (P/P_0_) increased from 0.45 to 0.99, a large hysteresis loop in the isotherm curves indicated the existence of mesopores. The pore size distribution of these samples is shown in Figure 8. The presence of mesopores in the range from 3 nm to 25 nm was clearly visible in both samples.

The porous structure of the carbon materials highly affects the adsorption processes and the amount of dye adsorbed on the surface. To determine the proper conditions of dyes adsorption, several factors have to be considered. In view of the properties of the used adsorbent, its surface area, porosity, and the functional groups on the surface are of the essence. This research aims at the determination of the influence of iron-doping and activator used on the ability of carbon materials to adsorb cationic (methylene blue) and anionic (congo red) dye.

**Figure 9:**
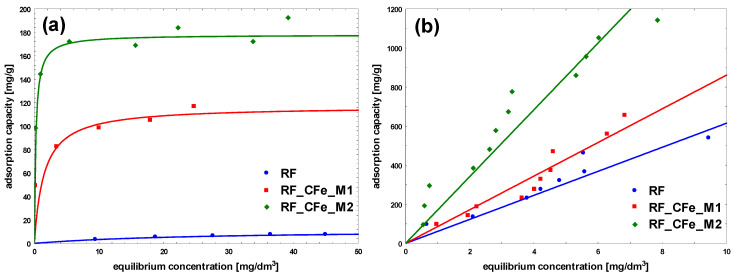
Adsorption isotherms on non-activated samples: (**a**) Methylene Blue, (**b**) Congo Red.

**Figure 10:**
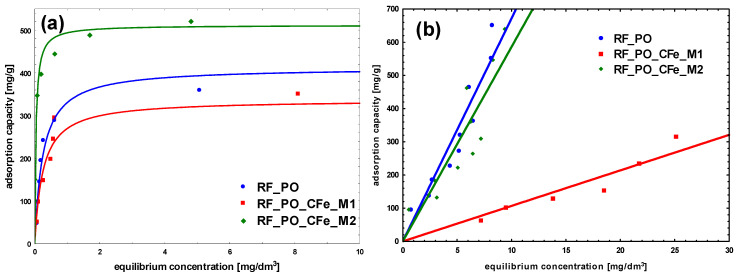
Adsorption isotherms on samples activated with potassium oxalate: (**a**) Methylene Blue, (**b**) Congo Red.

**Figure 11:**
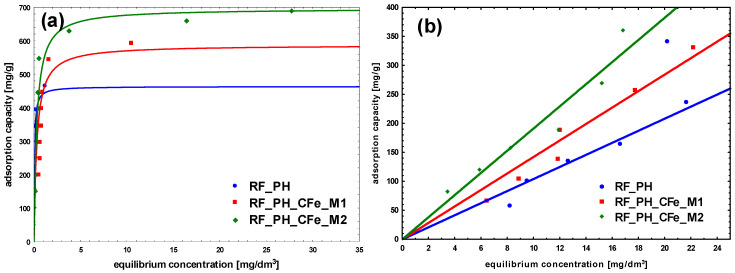
Adsorption isotherms on samples activated with potassium hydroxide: (**a**) Methylene Blue, (**b**) Congo Red.

To characterize the adsorption process between liquid and solid phases at the equilibrium state, the obtained adsorption isotherms were studied (Figure 9, Figure 10 and Figure 11). In the case of methylene blue, the adsorption equilibrium isotherms were found to follow the Langmuir isotherm model, assuming the presence of homogeneous binding sites with identical sorption energies on the surface of the adsorbent and without the interactions between adsorbed dye molecules [42].

The mathematic non-linear equation of the Langmuir isotherm can be written as follows:

(1) $$a=a_{m}\cdot \frac{b\cdot c}{1+b\cdot c}$$

where:

*a*—adsorption capacity [mg/g]

*a_m_*—maximum adsorption capacity of the monolayer of the adsorbent [mg/g]

*b*—Langmuir constant (equilibrium constant) [dm^3^/g]

*c*—equilibrium concentration MB or CR [mg/dm^3^]

In the case of congo red, an adsorption linear dependency of adsorption and concentration in a broad equilibrium concentration range has been noticed. Therefore, the adsorption process of this dye can be described by the Henry equation:

(2) a=H·c

where:

*a*—adsorption capacity [mg/g]

*H*—equilibrium constant [dm^3^/g]

*c*—equilibrium concentration MB or CR [mg/dm^3^]

The adsorption of methylene blue on carbon materials is heavily influenced by pore size. Mesopores enhance adsorption kinetics significantly, while micropores, although contributing to capacity, limit the rate of adsorption. Optimizing pore size distribution is a key to improve both the speed and effectiveness of dye-adsorption processes. Larger pore sizes provide less resistance and higher mobility for adsorbate molecules. To maximize adsorption efficiency, the pore size distribution of the adsorbent should be optimized to match the dimensions of the MB cation, ensuring sufficient mesopore content to facilitate effective dye adsorption [43,44]. The size of MB cation is about 1.5 × 1 × 0.5 nm [45]. For the reference non-activated sample, the lowest levels of the affinity of binding sites, as well as the maximum adsorption capacity of the monolayer was noticed. The highest value of b constant was obtained for the sample modified using M2 method, RF_CFe_M2 (Figure 9 and Table 2). Activation of carbon materials resulted in an improvement of the value of maximum adsorption capacity of the monolayer, which can be ascribed to the increased specific surface area of the material. The highest affinity of binding sites was calculated for the sample RF_PO_CFe_M2. Considering the values of the obtained equation constants, it can be stated that the sample RF_PO_CFe_M2 expresses considerably high sorption abilities towards MB. The highest value of b constant (27.5) from the Langmuir equation indicates strong interaction of MB with the surface of this adsorbent. KOH-activated samples have a very high mesoporous range and is the superior pore size for MB adsorption. Unfortunately, the adsorption capacity in these samples is lower than in samples activated with potassium oxalate. This is probably related to the structure of pores in these samples. The presence of H4-type hysteresis loops was confirmed in samples activated with potassium oxalate. The H4 hysteresis loop is indicative of narrow slit-like mesopores. In the samples activated with potassium hydroxide, a mixed type of hysteresis loop composed of H2- and H4-type was noticed, what suggests that the pore types are various, and that the distribution of pore diameter is relatively large, when the pore shape is neck-like, wide, or ink bottle-like. The movement of MB adsorbate in this type of pore may be restricted and more gradual. In the case of CR adsorption, the highest partition coefficient was noticed for RF_CFe_M2. These results suggest that the activation process affects the surface of the adsorbent. Congo red dye possesses hydrophilic sulfonate groups, and interacts strongly with the carbon surface containing a higher number of oxygen groups. On the other hand, MB particles prefer hydrophobic than hydrophilic active sites. 

It should be noted that for all experiments with congo red, the linear adsorption isotherms were recorded. It is possible that CR adsorption occurs in layers: after the formation of the first layer of adsorbed CR, the second one is formed on it, and so on. The CR molecule is symmetrical and has both anionic sulfonic groups and cationic amine groups. The adsorption can occur by binding the sulfonic or amine group to the sorbent surface. The remaining sulfonic or amine group can bind subsequent CR molecules. An increase of the concentration enhances the probability of the observed effect proportionally to the applied concentration.

## 3. Materials and Methods

### 3.1. Materials Preparation

The preparation of the reference, unmodified material called RF (as resorcinol—formaldehyde), as well as material activated using potassium oxalate RF 7/1 was described in our previous work [46]. Briefly, proper amounts of resorcinol and formaldehyde were mixed in an aqueous-alcohol solution maintaining proper pH value using ammonia water (25%). To activate the carbon material, potassium oxalate was applied and added to the solution before adding the formaldehyde. The mass ratio of potassium to carbon was 7 to 1. Next step of the preparation was the treatment in the microwave reactor and carbonization at 700 °C using the high-temperature tubular furnace.

To investigate the effect of different activators on the final properties of the obtained materials, potassium hydroxide was also used. However, the addition of KOH in the same preparation step as for potassium oxalate resulted in a too high pH value that prevented polycondensation. Therefore, the second activation method consisted of the addition of the 50 mL solution of potassium hydroxide to the resorcinol-formaldehyde resin gathered after the microwave treatment and mixing it for 24 h on a magnetic stirrer at ambient conditions. The weight ratio of potassium to carbon also equaled 7:1. The activated material was denoted as RF_PH. The rest of the preparation method was the same as described above.

The preparation of the iron-enhanced materials was described in our previous paper [32]. Two different methods were used to introduce iron citrate. In the first method (designated as M1), a proper amount of iron citrate was added to the solution of resorcinol in an aqueous alcohol solution, and next ammonia water and formaldehyde were added to carry out the resin polymerization. In the second method (designated as M2), the solution of iron citrate was first treated with ammonia water to precipitate iron hydroxide, and next, the resin polymerization was carried out.

### 3.2. Materials Characterization

The morphology of the obtained materials was investigated using the Apreo 2 Thermo Scientific Scanning Electron Microscope (Thermo Scientific, Waltham, MA, USA). TEM images were recorded using a FEI Tecnai F20 microscope (Tecnair, Shipley, UK). The phase composition was determined using X-ray diffraction method Cu Kα radiation (λCu Kα = 0.1540 nm) on an Empyrean (Panalytical, Malvern, UK). The identification of the crystalline phases was performed using HighScore+ and the ICDD PDF-4+ 2015 database.

The textural properties of the materials were evaluated using N_2_ adsorption/desorption on a QUADRASORB evoTM Gas Sorption automatic system (Quantachrome Instruments, Anton Paar Group AB, Boynton Beach, FL, USA) at −196 °C. The Brunauer–Emmett–Teller (BET) equation was used to determine the surface area (S_BET_), and S_BET_ was determined to be in the relative pressure range of 0.05–0.3. The total pore volume, TPV, was calculated from the volume of nitrogen held at the highest relative pressure (p/p0 = 0.99). The volume of micropores, V_m_, with dimensions less than 2 nm was calculated by integrating the pore volume distribution function using the DFT method; the mesopore volume, V_meso_, with dimensions from 2 to 50 nm, was calculated from the difference of the total pore volume, TPV, and the volume of micropores, V_m_. Before each adsorption experiment, the samples were outgassed at 250 °C under a vacuum of 1 × 10^−5^ mbar for 12 h using a MasterPrep multi-zone flow/vacuum degasser from Quantachrome Instruments (Anton Paar Group AB, Boynton Beach, FL, USA) to remove adsorbed species that could intervene in the adsorption processes.

The adsorption performance of produced composites relative to two kinds of commercial dyes (methylene blue and congo red) was determined. Methylene blue is a cationic water-soluble compound that is often used to estimate the removal capacity of porous materials. As the molecule size of methylene blue is >2 nm, the adsorption of this compound can indicate that the mesoporous structure of the material is favorable [47]. Congo red is an anionic dye largely produced and used in printing, textile, and dyeing industries. Its molecule size is similar to MB; then, a similar pore structure of an adsorbent would be useful.

### 3.3. Dye Solution Preparation

In a volumetric flask (1000 cm^3^), 1000 mg of dye was placed. Next, the distilled water was added to the flask up to the reference mark. Then, the magnetic stirrer was placed into the flask, and the whole was mixed for 1 h using a magnetic stirrer.

### 3.4. Adsorption Experiments

In the Erlenmeyer flasks, magnetic stirrers and 495 g of water were placed, and then 5 cm^3^ of a dye solution with a concentration of 1000 mg/dm^3^ was added, thus obtaining a solution with an initial concentration of C_0_ = 10 mg/dm^3^. Before the addition of carbon materials, the absorbance was measured for the initial concentration. The absorbance was measured in a glass cuvette with an optical path length of 1 cm using a Merc Pharo300 spectrophotometer at a wavelength of 664 nm for methylyne blue and 499 nm for congo red (Merck Millipore, Burlington, MA, USA). Then, carbon material was added to the flask in an amount of about 100 mg. Next, the flasks were placed on magnetic stirrers, and mixing was started. Measurements of absorbance were carried out every 24 h. After 24 h, the mixing was turned off, and after 2 h, the sample was centrifuged. The solution was then taken into the cuvette to determine the absorbance. Then, 5 cm^3^ of the dye solution was added to the flask, and the procedure was repeated. In the case when the absorbance value was higher than 1.8, 0.5 mL of the solution was taken from the flask and 3.5 mL of distilled water was added, thus obtaining an eightfold dilution.

Adsorption capacity [mg/g] was calculated by the following equation, where:

(3) $$a=V\frac{\left(C_{0}-C\right)}{m}$$

(4) $$C_{0}=\frac{V_{b}\cdot C_{b}}{V_{H_{2}O}+V_{b}}$$

*a*—adsorption capacity [mg/g];

*V*—total volume of the solution [dm^3^];

*C*_0_—initial concentration of dye [mg/dm^3^];

*C*—equilibrium concentration [mg/dm^3^];

*m*—the mass of the adsorbent [g];

*V_b_*—volume of the dye solution (5 mL) [dm^3^];

*C_b_*—the concentration of the dye solution (1000 mg/dm^3^) [mg/dm^3^];

$V_{H_{2}O}$—volume of distilled water solution (495 mL) [dm^3^].

The tests were carried out for the following initial dye concentration values: 10; 20; 29; 39; 48; 57; 66; 75; 83 and 92 mg/dm^3^.

## 4. Conclusions

The influence of modification with iron citrate as well as the activation using potassium oxalate and potassium hydroxide on the adsorption properties of carbon spheres was studied. It was found that in the case of methylene blue, the adsorption equilibrium isotherms followed the Langmuir isotherm model, assuming the presence of homogeneous binding sites with identical sorption energies on the surface of the adsorbent and without the interactions between adsorbed dye molecules. The adsorption performance of methylene blue on carbon materials strongly depended on porous structure. The adsorption capacity in the samples activated with potassium hydroxide was lower than in the case of the samples activated with potassium oxalate, what can be related to the structure of pores (more neck-like or ink bottle-like). In the case of congo red, a linear dependency of adsorption and concentration in a broad-equilibrium concentration range was found and well-described using the Henry equation. It was stated that congo red dye possesses hydrophilic sulfonate groups interacted strongly with the carbon surface containing a higher number of oxygen groups. The most efficient adsorption of methylene blue was noticed for the sample activated with potassium hydroxide and modified with iron citrate, and a maximum adsorption capacity of 696 mg/g was achieved. The highest congo red adsorption was noticed for the non-activated sample modified with iron citrate, and partition coefficient for this material equaled 171 dm^3^/g. It is worth noting that in both cases, the direct introduction of iron citrate into the resorcinol/alcohol solution worsens the adsorption properties of the obtained materials. It should be underlined that precipitation of iron hydroxide before the formation of resorcinol-formaldehyde resin led to obtaining materials with better adsorption properties in comparison with the materials where iron citrate was added during the formation of the resin. It can be related to an increase of mesopores contribution. The presence of iron carbide or/and metallic iron in the obtained materials could be beneficial with regard to the further magnetic separation of the adsorbents from liquid phase.

## Figures and Tables

**Table 1:** Textural parameters for the obtained samples [32].

	S_BET_ [m^2^/g]	TPV [cm^3^/g]	V_m_ (<2 nm) [cm^3^/g]	V_meso_ [cm^3^/g]
RF	455	0.26	0.22	0.04
RF_CFe_M1	400	0.33	0.13	0.20
RF_CFe_M2	439	0.39	0.14	0.25
RF_PO	942	0.53	0.44	0.07
RF_PO_CFe_M1	826	0.47	0.37	0.10
RF_PO_CFe_M2	656	0.48	0.27	0.21
RF_PH	1473	0.83	0.67	0.16
RF_PH_CFe_M1	991	0.78	0.39	0.39
RF_PH_CFe_M2	974	1.01	0.37	0.64

**Table 2:** Adsorption isotherm coefficients.

		Methylene Blue		Congo Red	
	*a_m_*, mg/g	*b*, dm^3^/g	*R* ^2^	*H*, dm^3^/g	*R* ^2^
RF	12	0.04	0.99	61.5	0.98
RF_CFe_M1	117	0.67	0.94	86.1	0.98
RF_CFe_M2	178	4.30	0.91	171.0	0.98
RF_PO	413	4.0	0.92	67.2	0.98
RF_PO_CFe_M1	337	4.0	0.91	10.7	0.98
RF_PO_CFe_M2	492	31.5	0.98	58.6	0.95
RF_PH	463	20.3	0.97	10.4	0.99
RF_PH_CFe_M1	587	3.3	0.90	14.2	0.99
RF_PH_CFe_M2	696	3.4	0.95	19.1	0.99

## Data Availability

The original contributions presented in the study are included in the article, further inquiries can be directed to the corresponding authors.
